# Localized nonlinear excitations in diffusive memristor-based neuronal networks

**DOI:** 10.1371/journal.pone.0214989

**Published:** 2019-06-04

**Authors:** A. S. Tankou Tagne, C. N. Takembo, H. G. Ben-Bolie, P. Owona Ateba

**Affiliations:** 1 Laboratory of Nuclear Physics, Department of Physics, Faculty of Science, University of Yaounde I, Cameroon; 2 Laboratory of Biophysics, Department of Physics, Faculty of Science, University of Yaounde I, Cameroon; Lanzhou University of Technology, CHINA

## Abstract

We extend the existing ordinary differential equations modeling neural electrical activity to include the memory effect of electromagnetic induction through magnetic flux, used to describe time varying electromagnetic field. Through the multi-scale expansion in the semi-discrete approximation, we show that the neural network dynamical equations can be governed by the complex Ginzburg-Landau equation. The analytical and numerical envelop soliton of this equation are reported. The results obtained suggest the possibility of collective information processing and sharing in the nervous system, operating in both the spatial and temporal domains in the form of localized modulated waves. The effects of memristive synaptic electromagnetic induction coupling and perturbation on the modulated action potential dynamics examined. Large electromagnetic induction coupling strength may contribute to signal block as the amplitude of modulated waves are observed to decrease. This could help in the development of a chemical brain anaesthesia for some brain pathologies.

## Introduction

Even after over a century of active reported investigations, the human brain made up millions of inter-connected neurons continue to reveal new complexities in behavior due to its complex dynamics. Based on the seminal work by Hodgkin and Huxley (HH) [1] linked to electrophysiological experiments, the form of the signal carrying information in the nervous system is nowadays well accepted as an impulse, originating from the potential difference across the cell membrane. The biological HH neuron model and many of its simplified versions have confirmed their effectiveness for recognizing and understanding the electrical activities in neurons. Among them we cite the FitzHugh Nagumo (FHN) [2, 3], Hindmarsh Rose (HR) [4–6], Morris Lecar (ML) [7], Izhikevich Model [8] etc.

The electrical activity of biological cell membrane can be altered when exposed to electromagnetic induction, created during the period of ions current exchange as well as during fluctuation in ion concentration [9–11]. Following this report, many improved versions of the neurons model had been proposed. Using these models, the social concern of external electromagnetic radiation from the increasing usage of mobile communication on human health has been examined [12]. Takembo *et al*. [13] reported the possibility of conduction block in myocardial tissue when are exposed to continuous electromagnetic field. *Ma et al*. [10, 14] proposed two death mechanism associated with increasing electromagnetic exposure. Mvogo *et al*. [15] showed diverse spatiotemporal patterns in the neuronal networks under electromagnetic induction radiation. Thus it was generally observed that the electrical activity and oscillating behaviors of biological cells are greatly reduced due to electromagnetic radiation exposure and which is consistent with biological experiments [16, 17].

The difference in membrane potentials between adjacent neurons in a network can induce an electromagnetic induction current that behaves like a memristive synaptic coupling thereby uniting and ensuring the collective dynamics of the networks. Using a memristive electromagnetic coupling between two adjacent neurons, Bao *et al*. [18] reported the existence of interesting multiple firing patterns. Xu *et al*. [19] used the memristor to connect two neurons and the phase synchronization in electrical activities discussed. It is found that synchronization and pattern stability can be enhanced under memristive coupling. Through the mechanism of modulational instability, Takembo *et al*. [20] discussed the possibility of global synchronization for small memristive synaptic coupling within a neuronal network with nearest neighbor interaction. Albeit the satisfactory results discussed, the appropriate mechanism underlying the spatial structures of activity within a reliable neuronal network is still under investigation. Analyzing the mechanisms governing spatial structures of electrical activity in neuronal network is vital in understanding a wide range of both naturally occurring and pathological phenomena [21, 22].

This work is motivated by experimental and theoretical investigations, which under proper assumptions have reported the existence of localized structures within the specific population of connected neurons [23, 24]. Determining the onset of cortical waves propagation within the brain is fundamental to comprehend the normal processing of information as well as some pathological manifestations including migraines, epileptic seizures [25].

Consequently, this paper main objective is to study via both analytical and numerical methods, the dynamics of modulated waves in a diffusive memristor-based neuronal networks. We apply the multiple scale expansion method in the semi-discrete approximation to come out with a modified complex Ginzburg-Landau (CGL) equation by means of a specific perturbation technique. From there, we present the envelope soliton solution of the CGL to obtain an expression of the nerve impulse. Finally, we present the role of memristive synaptic electromagnetic induction coupling and effect of small perturbation on the dynamics of the envelope soliton solution. It is found that the memristive synaptic electromagnetic induction current could contribute to conduct block during modulated wave propagation.

## Materials and methods

### Model setting and perturbation technique

The FHN model is a refined HH model, which is itself a generalization of the Van der Pol(VDP) oscillator. This model was proposed on the assumption that the neural medium implies some periodic oscillations. The FHN model is today used as a generic model of excitability and oscillatory dynamical behavior. It has been greatly contributing in nonlinear neurodynamical domain and has become prototype model for systems exhibiting excitability. The FHN model is governed by a set of two nonlinear ordinary differential equations on the dimensionless variables *x*(*t*) and *y*(*t*). The variable *x*(*t*) represents the membrane potential (nerve impulse). The dynamics of a single neuron is then describe by

$$\begin{array}{c} \begin{array}{cc} \frac{dx}{dt} & =\frac{1}{a}\left(x-\frac{x^{3}}{3}-y+I_{ext}\right),\\ \frac{dy}{dt} & =ax-by+c. \end{array} \end{array}$$ (1)

*I*_*ext*_ is the stimulation current. The rest parameters are carefully chosen so as to reproduce the main characteristics of excitable medium. To be consistent with previous works on the model, we selected *a* = 0.15, *b* = 0.2 and *c* = 0.3. As review in ref. [20], the slow parameter *y*(*t*) could be interpreted as the magnetic flux variable from Maxwell electromagnetic theory (*y* ≡ *ϕ*). The new improved FHN model for *M*-neurons, in which each neuron is coupled to its nearest neighbor through the memristive synaptic electromagnetic induction current with strength *K*. The new model could reproduce the same dynamical behavior observed in the original FHN and in addition, expands the bifurcation parameter regions [26]. The new dynamical equations are given by

$$\begin{array}{c} \begin{array}{cc} \frac{dx_{m}}{dt} & =\frac{1}{a}\left(x_{m}-\phi_{m}-\frac{x_{m}^{3}}{3}+I_{ext}\right)+K\rho \left(\phi_{m}\right)\left(x_{m+1}-2x_{m}+x_{m-1}\right),\\ \frac{d\phi_{m}}{dt} & =ax_{m}-b\phi_{m}+c, \end{array} \end{array}$$ (2)

with $\rho \left(\phi_{m}\right)=\alpha +3\beta \phi_{m}^{2}$ and *m* = 1, …, *M*. The term *ρ*(*ϕ*_*m*_) represents the memductance of the memristor, used to depict the modulation of time-varying electromagnetic field on the gap junction membrane. Many authors have investigated wave propagation via pattern formation in various improved excitable systems. For example, Qian *et al*. [27] systematically analyzed the role of network topology and other system parameters on the spatiotemporal dynamics in excitable homogeneous random networks. Qu *et al*. [28] reported irregular mixed-mode oscillation due to the influence of stochastic electromagnetic disturbance autaptic neuronal network. Takembo *et al*. [29] reported the possibility of achieving perfect intercellular communication in memristor-based neuronal network using a controlled pitch of electromagnetic radiation. However, the results obtained through numerical experiments above present complex dynamical structures attributed to the large number of tilted waves excited and which compete with others.

In this paper, we make use of the semi-discrete approximation to derive analytically the type of localized waves that propagate in diffusive memristor-based neuronal networks. In order to successfully apply this method, we firs transform Eq (1) into the wave form. By differentiating *x*_*m*_ with respect to time and substituting in ${\dot{x}}_{m}$ yields the governing equation

$$\begin{array}{c} \begin{array}{cc} \ddot{x_{m}}+\Omega_{0}^{2}x_{m}+\gamma_{0}\phi_{m}^{2}+\gamma_{1}x_{m}^{3}+\gamma_{2}x_{m}^{2}{\dot{x}}_{m}+I_{0}=K_{0}\left(x_{m+1}-2x_{m}+x_{m-1}\right) & \\ +K_{1}\left({\dot{x}}_{m+1}-2{\dot{x}}_{m}+{\dot{x}}_{m-1}\right)+K_{2}\left(x_{m+1}-2x_{m}+x_{m-1}\right)\phi_{m}{\dot{x}}_{m} & \\ +K_{3}\left(x_{m+1}-2x_{m}+x_{m-1}\right)\phi_{m}^{2}+K_{4}\left({\dot{x}}_{m+1}-2{\dot{x}}_{m}+{\dot{x}}_{m-1}\right)\phi_{m}^{2}, \end{array} \end{array}$$ (3)

$$\begin{array}{c} \dot{\phi_{m}}+b\phi_{m}-ax_{m}=0, \end{array}$$ (4)

where $\Omega_{0}^{2}=1-a^{-2}$, $\gamma_{0}=\frac{1}{a}\left(1-b\right)$, $\gamma_{1}=\frac{1}{3a^{2}}$, $\gamma_{2}=\frac{1}{a}$, *K*_0_ = *αK*, *K*_1_ = *K*_2_ = *αK*,*K*_3_ = 6*βK* and *K*_4_ = 3*βK*_4_.

Nonlinear dynamical equations owing to their complexity, are typically not accessible to analytical methods of problem resolution. It is possible to obtain nearly exact solution through a special perturbation technique. In addition since we are looking for solution in a weakly dissipative environment, we introduce the perturbation

$$\begin{array}{c} \left(\gamma_{0},\gamma_{1},\gamma_{2},K_{1},K_{2},K_{3},K_{4}\right)\to \epsilon^{2}\left(\gamma_{0},\gamma_{1},\gamma_{2},K_{1},K_{2},K_{3},K_{4}\right), \end{array}$$ (5)

with *ϵ* being a small perturbation parameter value. The equations become

$$\begin{array}{c} \begin{array}{cc} \ddot{x_{m}}+\Omega_{0}^{2}x_{m}+\epsilon^{2}\left(\gamma_{0}\phi_{m}^{2}+\gamma_{1}x_{m}^{3}+\gamma_{2}x_{m}^{2}{\dot{x}}_{m}\right)+I_{0}=K_{0}\left(x_{m+1}-2x_{m}+x_{m-1}\right) & \\ +\epsilon^{2}K_{1}\left({\dot{x}}_{m+1}-2{\dot{x}}_{m}+{\dot{x}}_{m-1}\right)+\epsilon^{2}K_{2}\left(x_{m+1}-2x_{m}+x_{m-1}\right)\phi_{m}{\dot{x}}_{m} & \\ +\epsilon^{2}K_{3}\left(x_{m+1}-2x_{m}+x_{m-1}\right)\phi_{m}^{2}+\epsilon^{2}K_{4}\left({\dot{x}}_{m+1}-2{\dot{x}}_{m}+{\dot{x}}_{m-1}\right)\phi_{m}^{2}, \end{array} \end{array}$$ (6)

$$\begin{array}{c} \dot{\phi_{m}}+b\phi_{m}-ax_{m}=0. \end{array}$$ (7)

The above system of equations are therefore those regulating the dynamics of the localized excitation in the new FHN neural model. We are interested in studying low-amplitude nonlinear excitations in a weakly diffusive neural network. We therefore use the multiple scale expansions in semi-discrete approximation [30, 31] to obtain the CGLE.

We proceed, by considering a slowly varying carrier wave envelope with angular frequency(*ω*) and wave number(q). Using the change of variables *x*_*m*_ = *ϵ*(*m* − *c*_*g*_*t*) and *τ* = *ϵ*^2^*t*, we use the trial solution;

$$\begin{array}{c} \begin{array}{c} x_{m}\left(t\right)=A_{1}\left(\xi ,\tau \right)e^{i\theta_{m}}+\epsilon A_{0}\left(\left(\xi ,\tau \right)\right)+\epsilon A_{2}\left(\xi ,\tau \right)e^{2i\theta_{m}}+c.c+0\left(\epsilon^{2}\right),\\ \phi_{m}\left(t\right)=B_{1}\left(\xi ,\tau \right)e^{i\theta_{m}}+\epsilon B_{0}\left(\xi ,\tau \right)+\epsilon B_{2}\left(\xi ,\tau \right)e^{2i\theta_{m}}+c.c+0\left(\epsilon^{2}\right), \end{array} \end{array}$$ (8)

with the wave phase *θ*_*m*_ = *qm* − *ωt*, c.c the complex conjugate and *c*_*g*_ being the group velocity whose expression will be determined later.

## Results and discussion

### Damped Ginzburg-Landau equation

Replacing Eq (8) into system of Eqs (6) and (7), we obtained at the order $\epsilon^{0}\times e^{i\theta_{m}}$ for *x*_*m*_(*t*), the dispersion relation

$$\begin{array}{c} \begin{array}{c} \omega^{2}=\Omega_{0}^{2}+2K_{0}\left(1-\text{cos}\left(q\right)\right). \end{array} \end{array}$$ (9)

With reference to the original model parameters, Ω_0_ and *K*_0_ are positive constants. Eq (9) is valid for wave number

$$\begin{array}{c} q\geq q_{c}=arccos(1-\frac{\omega^{2}-\Omega_{0}^{2}}{2K_{0}}). \end{array}$$ (10)

It follows from Eq (10) that the improve model is a pass band filter, allowing waves to propagate in the network with frequency in the domain [*ω*_*ci*_, *ω*_*cf*_], where the square of the lower and upper cut-off frequencies are given by $\omega_{ci}^{2}=\Omega_{0}^{2}$ and $\omega_{cf}^{2}=\Omega_{0}^{2}+4K_{0}$. We recall $\Omega_{0}^{2}=\alpha K$, indicating that the lower and upper cut-off frequencies depends on the electromagnetic induction coupling parameters *α* and *K*_0_. This is very important from biophysical point of view. It suggests the effect of electromagnetic induction could effectively change the collective dynamics of neuronal network by altering the frequency of oscillation which is fundamental in the information processing in the nervous system.

**Fig 1:**
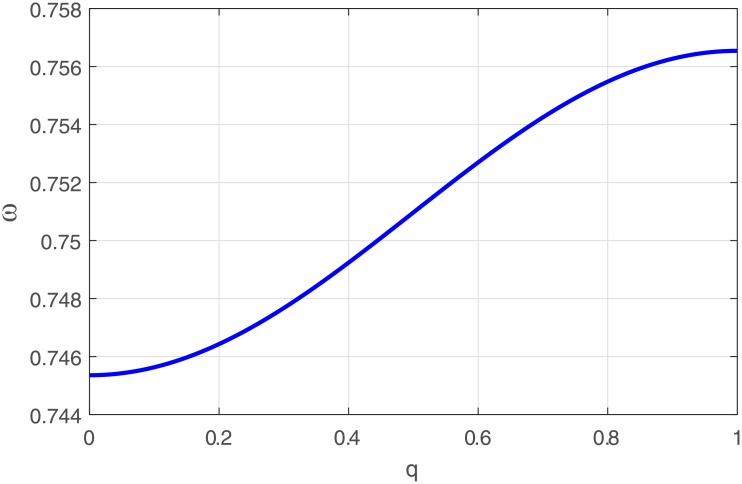
Panel displays the plot of angular frequency, vs. the wave number, given by the dispersion relation in Eq (9) for *K*_0_ = 0.021.

**Fig 2:**
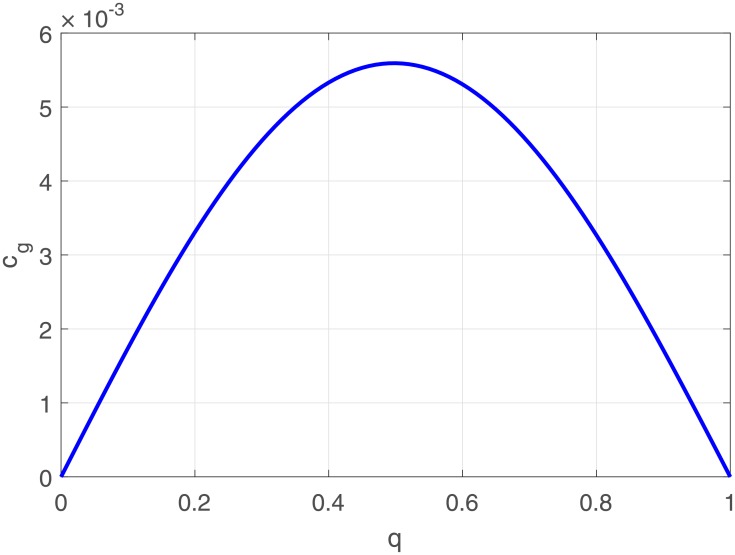
Panel depicts the plot of group velocity vs. the wave number for *K*_0_ = 0.021.

We plot the angular frequency(*ω*) versus the wave number(q) [Fig 1]. This plot is similar to that of the pass band filter, with corresponding lower and upper cut-off frequencies. The influence of feedback gain through the memristor coupling is clearly depicted in Fig 2 to be a decreasing function of the angular frequency. As the memristor coupling is increased, the lower cut off frequency is observed to be lowered.

At the same order $\epsilon^{0}\times e^{i\theta_{m}}$, Eq (7) for *ϕ*_*m*_(*t*) gives the relation

$$\begin{array}{c} \begin{array}{c} B_{1}=\left(a_{1}+ia_{2}\right)A_{1}, \end{array} \end{array}$$ (11)

with

$$\begin{array}{c} \begin{array}{c} a_{1}=\frac{ab}{b^{2}+\omega^{2}},a_{2}=\frac{a\omega }{b^{2}+\omega^{2}}. \end{array} \end{array}$$ (12)

At the order $\epsilon^{1}\times e^{i\theta_{m}}$, we obtain the group velocity relation;

$$\begin{array}{c} c_{g}=\frac{K_{0}\text{sin}\left(q\right)}{\omega }. \end{array}$$ (13)

A plot of the group velocity relation is clearly revealed in Fig (2). Finally at the order $\epsilon^{2}\times e^{i\theta_{m}}$, while making use of the previous relations, we obtain

$$\begin{array}{c} j\frac{\partial A_{1}}{\partial \tau }+P\frac{\partial^{2}A_{1}}{\partial \xi^{2}}+Q{\left|A_{1}\right|}^{2}A_{1}+jRA_{1}=0, \end{array}$$ (14)

where

$$\begin{array}{c} \begin{array}{c} P=\frac{K_{0}\omega^{2}\text{cos}\left(q\right)-c_{g}^{2}}{2\omega }, \end{array} \end{array}$$ (15)

$$\begin{array}{c} \begin{array}{c} Q=Q_{1}+jQ_{2}, \end{array} \end{array}$$ (16)

$$\begin{array}{c} R=2K_{1}{\text{sin}}^{2}\left(\frac{q}{2}\right). \end{array}$$ (17)

*Q*_1_ and *Q*_2_ represent the real and imaginary parts of Q, the nonlinearity coefficient given by

$$Q_{1}=-\frac{3\gamma_{1}}{2}-a_{1}a_{2}K_{2}\omega^{2}-\frac{K_{3}\omega }{2}\left(3a_{1}^{2}+a_{2}^{2}\right)+4a_{1}a_{2}K_{4}{\text{sin}}^{2}\left(\frac{q}{2}\right),$$ (18)

$$Q_{2}=\frac{\gamma_{1}}{2}-\frac{K_{2}\omega^{2}}{2}\left(a_{1}^{2}-a_{2}^{2}\right)-a_{1}a_{2}K_{2}\omega +2K_{4}\left(a_{1}^{2}+3a_{2}^{2}\right){\text{sin}}^{2}\left(\frac{q}{2}\right).$$ (19)

Eq (14) is the Complex Ginzburg-Landau Equation(CGLE) describing the evolution of modulated waves in our memristor-based neuronal network. The CGLE and many of its modified versions have been very useful in the study of many physical phenomena [32]. This include the domain of nonlinear optics and Bose-Einstein condensation. In addition, Eq (14) is a spatiotemporal equation which therefore suggests an important biophysical significance. It supports the suggestion that neurons can effectively participate in the collective long-scale information processing in the brain in both the space and the time domains. The CGLE has been proposed recently in the study of nonlinear myocardial impulses in the diffusive magnetic myocardial model [13, 33].

It is essential to point out that in some physical contexts when the dissipation is neglected, the evolution equation is reduced to the Nonlinear Schrödinger Equation [34]. This is not the case in the present context since the nonlinearity is complex. Thus the evolution of action potential in our improved model will always be modeled by the CGLE.

**Fig 3:**
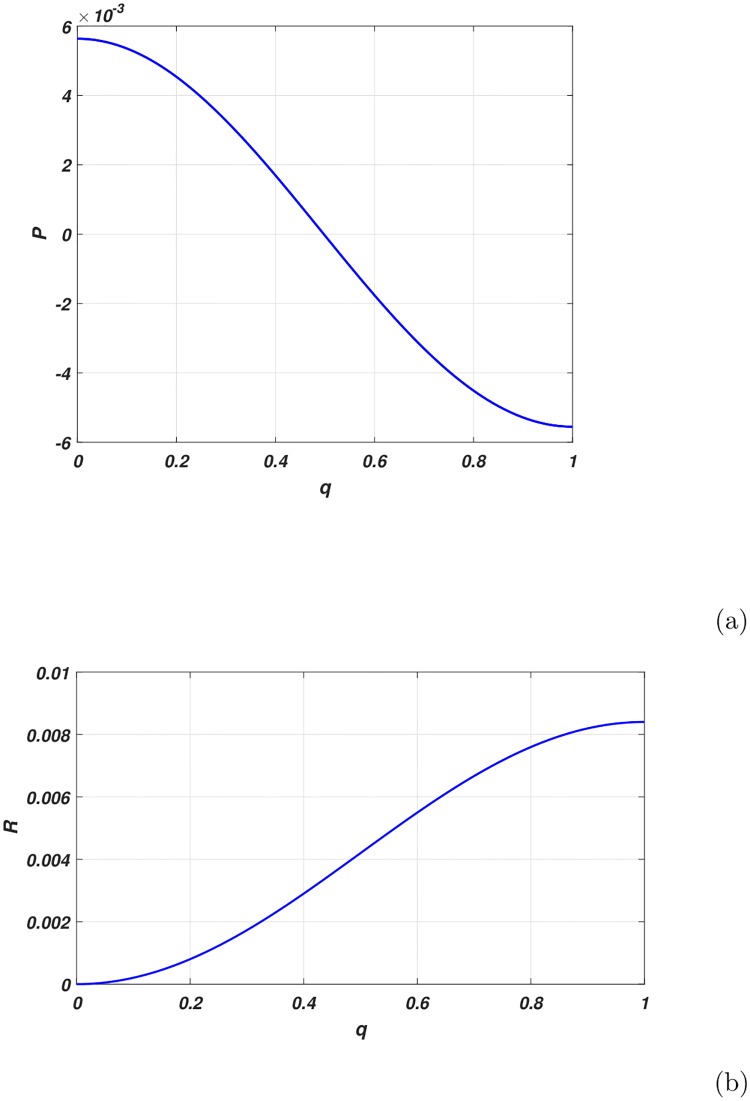
Variation of P [Fig 3(a)] and R [Fig 3(b)] in terms of the wave number of the carrier wave.

**Fig 4:**
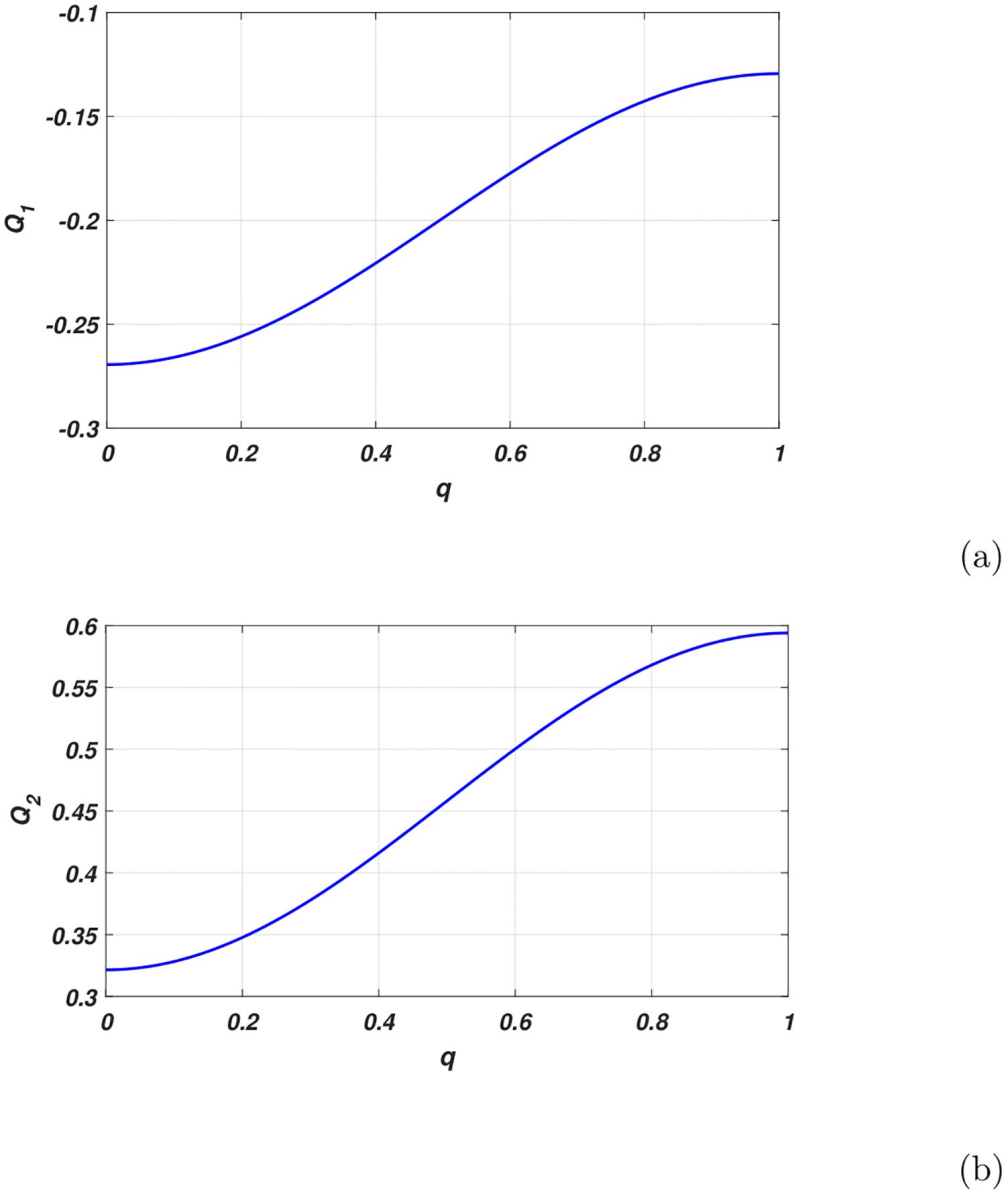
Variation of *Q*_1_[Fig 4(a)] and *Q*_2_[Fig 4(b)] in terms of the wave number of the carrier wave.

**Fig 5:**
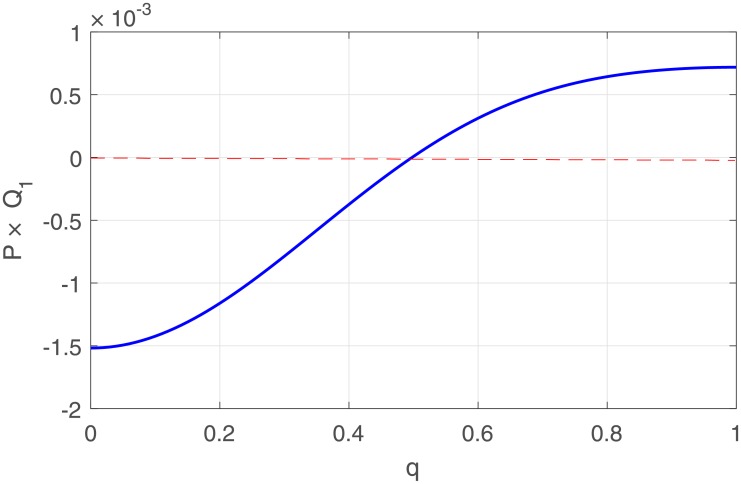
Variation of *P* × *Q*_1_ in terms of the wave number of the carrier wave.

The variation of *P* and *R* versus the wave number are depicted in Fig 3(a) and 3(b). Whereas *Q*_1_ and *Q*_2_ in Fig 4(a) and 4(b). and *P* × *Q*_1_ in Fig 5. We observed from the plots *Q*_1_ is strictly negative while *Q*_2_ is strictly positive.

We remark as well that P is real thus the modulational instability will depends on the sign of the product *P* × *Q*_1_. According to Benjamin-Feir instability analysis, plane waves are stable for negative values of *P* × *Q*_1_ whilst they are unstable for positive values. Spatially localized nerve impulse is therefore expected from the diffusive memristor-based neuronal networks for carrier wave whose wave number positive domain as defined by Fig 5. In the next section we calculate and plot the nonlinear solution of the equation of motion.

### Nonlinear solutions of the modified CGLE

The modified CGLE obtained above does not govern the spatiotemporal evolution of the nerve impulse, but rather of the amplitude of one term in the series representing the overall solution to Eqs (6) and (7). To calculate the general solution representing the wave form associated with the nerve impulse in our model, we proceed as follows. We rewrite Eq (14) in the form

$$\begin{array}{c} i\frac{\partial A_{1}}{\partial \tau }+P\frac{\partial^{2}A_{1}}{\partial \xi^{2}}+Q_{1}|A_{1}{|}^{2}A_{1}=-i(Q_{2}|A_{1}{|}^{2}+R)A_{1}. \end{array}$$ (20)

The left hand side of Eq (20) corresponds to the well known Nonlinear Schrödinger (NLS) equation whose exact solution is well known, depending on the sign of *P* × *Q*_1_. When *P* × *Q*_1_ is strictly positive, the solution obtained corresponds to envelope soliton. Several solitonic solutions for different classes of NLS equation and many other nonlinear evolution equations have been investigated [35–40]. The general solution of the left hand side of Eq (20) is given by

$$\begin{array}{c} A_{1}\left(\xi ,\tau \right)=A_{0}sech\left[A_{0}{\left(\frac{Q_{1}}{P}\right)}^{\frac{1}{2}}\xi \right]e^{i(\frac{Q_{1}A_{0}^{2}}{2})\tau } \end{array}$$ (21)

The solution of Eq (20) can then be obtained by using the transformation

$$\begin{array}{c} F\left(\xi ,\tau \right)=A_{1}\left(\xi ,\tau \right)e^{i(\sigma \tau )}, \end{array}$$ (22)

where *A*_1_(*ξ*, *τ*) is the general solution of NLSE, given by Eq (21), with

$$\begin{array}{c} \sigma =-i(Q_{2}|A_{1}{|}^{2}+R). \end{array}$$ (23)

We finally write the solution as

$$\begin{array}{c} \begin{array}{cc} x_{m}\left(t\right)= & 2X_{0}sech\left[X_{0}{\left(\frac{Q_{1}}{P}\right)}^{\frac{1}{2}}\left(m-c_{g}t\right)\right]\text{cos}\left[qm+\left(\frac{Q_{1}X_{0}^{2}}{2}-\epsilon^{2}\omega \right)t\right]\\ & \times \text{exp}\left[-\left(\frac{\epsilon^{2}R}{2}+Q_{2}X_{0}^{2}sech^{2}\left[X_{0}{\left(\frac{Q_{1}}{P}\right)}^{\frac{1}{2}}\left(m-c_{g}t\right)\right]\right)\right]+0\left(\epsilon^{2}\right) \end{array} \end{array}$$ (24)

with *X*_0_ = 2*A*_0_.

**Fig 6:**
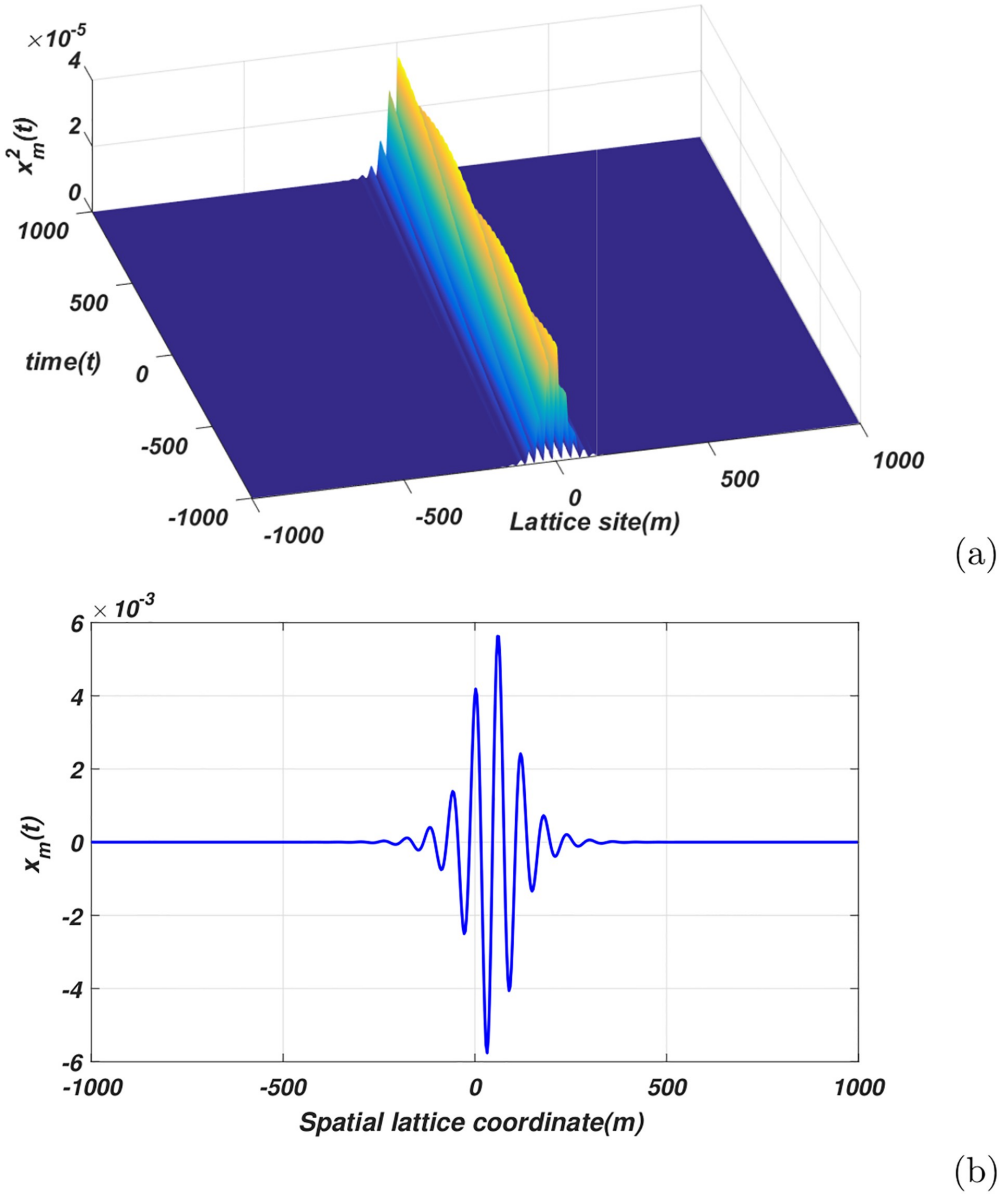
(a) Profile of analytical envelope solution of Eq (14)[Fig 6(a)], (b) The spatial feature of the analytical solution, for *t* = 10000.0, *A*_0_ = 0.0015 and *ϵ* = 0.08[Fig 6(b)].

Fig 6(a) and 6(b) portray the depiction and behavior the ionic wave in the main computational domains. Thus Eq (24) represents a breathing soliton [Fig 6(b)] displaying, two vital physical features; hyperpolarization and the refractory periods, observed in experiments. In Fig 6(b), the undershoot characterizing the two features is manifested through a phenomenon by which part of the nerve input signal goes below zero. In addition, as reviewed in ref. [41], the localized structures have all the features of the nerve impulses. They carry information from one node to another for a better coordination of some important neural processes on one side, and in pathological situations in another regard.

**Fig 7:**
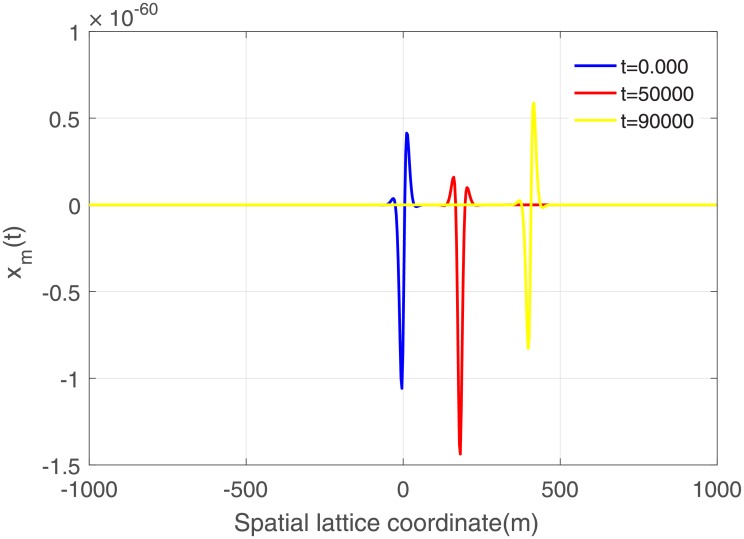
Profile of spatial envelope solution of Eq (14) at different time for *ϵ* = 10.00 and *A*_0_ = 0.009.

We performed numerical simulation of the CGLE [Eq (14)] via Runge-Kutta method with fixed step size. For the initial condition taken as Eq (14), the profile of the signal is presented in Fig 7. As expected the solution of the equation is an envelope soliton. The snapshots of the lattice at several distinct times reveal the fact that the envelope is structurally unstable, as it changes its shape in the course of its propagation through the lattice. Indeed, this asymmetric solution is indicative of the presence of a bright envelope soliton propagating within the one-dimensional spatial network of neurons, and whose amplitude is correspondingly depicted.

**Fig 8:**
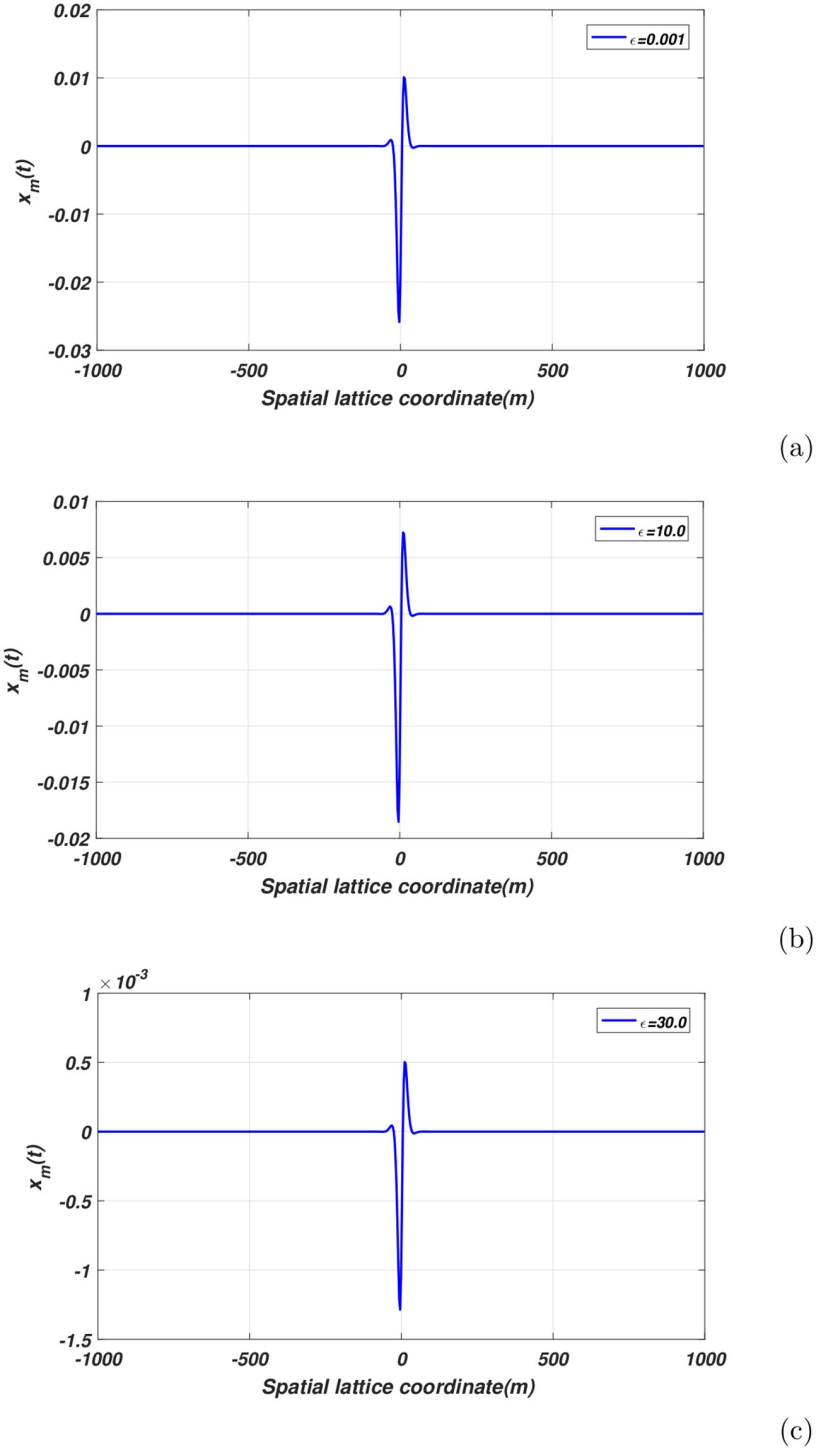
Profile of spatial envelope solution at different perturbation *ϵ* for *t* = 0.0, *q* = 0.7*π* and *A*_0_ = 0.009[Fig 8(a)-(c)].

Furthermore, it is vital to note that the contribution of the perturbation parameter *ϵ* in helping to differentiate the orders of development of the solitonic pulse. This effect on the nerve wave can be seen in Fig 8(a)–8(c). We can observe the variation of the perturbation parameter *ϵ* affects only the amplitude and not the form of the nerve wave. Indeed, an increase in *ϵ* is characterized by a corresponding increased in the random motion of ions exchanged across the plasma membrane. This reduces the width of the propagating action potential thereby increasing the amplitude.

**Fig 9:**
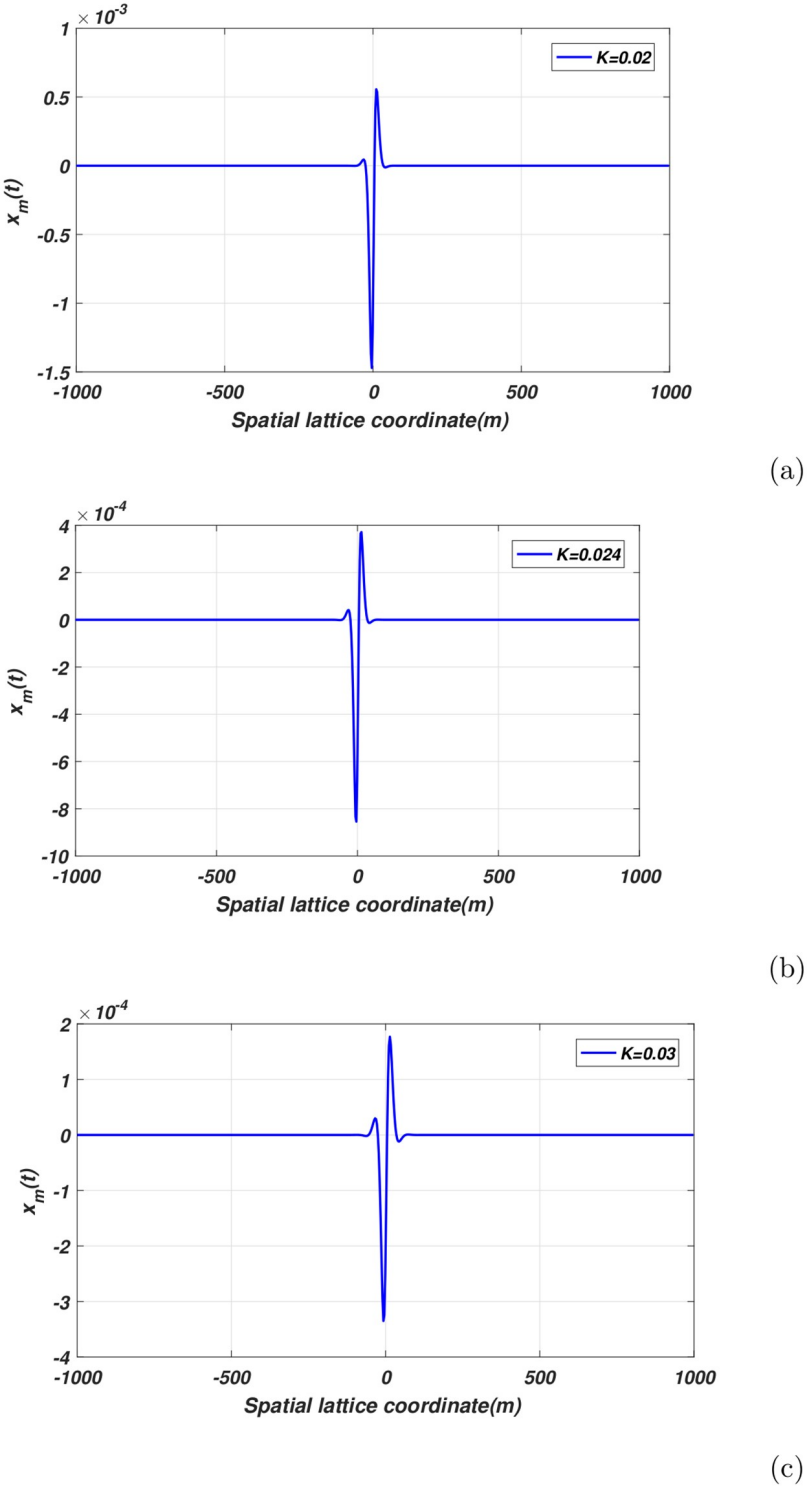
Profile of spatial envelope solution at different memristive synaptic electromagnetic coupling *K* for *t* = 0.0, *q* = 0.7*π* and *A*_0_ = 0.009[Fig 9(a)-(c)].

The impact of the electromagnetic induction current coupling *K* are illustrated in Fig 9(a)–9(c). Increasing the parameter *K* decreases the nerve wave amplitude. The result of solution (24) is the phenomenon of nerve-impulse blockage, associated with an increased of the electromagnetic induction current coupling. As one can see, the breathing soliton is highly sensitive to changes in *K*. Once more, the possible mechanism could be the polarization and magnetization of the neuronal network resulting in the impulse signal becoming completely damped. This phenomenon, reflecting a blockage of the nerve signal due to changes in the memristive synaptic electromagnetic induction current coupling, is very useful in the development of chemical anesthesia. Nerve-signal blockage is not a new context but has been observed and reported in several models of the biological neuron. Shneider *et al*. [42], use the Hodgkin-Huxley model, to show that an increase in the perturbation amplitude could initiate the action potential. Nevertheless, a further increase may cause blockage of the nerve impulse in a region of depleted channel density. In addition, Novacek *et al*. [43] using the Fitzhugh-Nagumo neuron model in a numerical way showed that an application of high-frequency stimulations on the neuron could lead to impulse blockage, whereas low frequency simulations of the nerve would favor propagation of the nerve impulse.

## Conclusion

The main goal of this paper was to study nonlinear localized excitations in an improved FitzHugh-Nagumo (FHN) neural networks where adjacent cells are connected through memristive synaptic electromagnetic induction current coupling with nearest neighbor interactions. By transforming the new model dynamical equations into wave form, we proceed to find low amplitude modulated wave solution of the networks. To achieve this, we apply the multiple scale analysis in the semi-discrete approximation. We obtain at the first and second order, the dispersion and group velocity relations of the stimulus dependent on the memristive electromagnetic induction current coupling and other system parameters. At the third order of approximation, we obtain a modified Complex Ginzburg-Landau equation (CGLE) from the transmembrane potential equation of motion, which is an equation governing the evolution of modulated waves in the networks. It confirms that neurons can effectively participate in the collective long-scale information processing in the brain in both the space and the time domains. By direct resolution of the obtained CGLE, the analytical solution portrays an asymmetric envelope soliton with features of impulses. This modulated soliton properties are showed to be greatly influenced by electromagnetic induction coupling as well as the impact of small perturbation.
